# Supplementary material for: Content-rich biological network constructed by mining PubMed abstracts
Source: BMC Bioinformatics. 2004 Oct 8;5:147. doi: 10.1186/1471-2105-5-147 (PMC528731; doi:10.1186/1471-2105-5-147)
Supplement: Additional File 2 — The original results of the above study (non-essential files are deleted to keep the file size under the limit set by BMC bioinformatics). [file 1471-2105-5-147-S2.bz2 › chilibotAdditionalFile2/dip05/749ID10485884E18132/html/BTUB_TONB.html]

 


 **BTUB** and **TONB** 
  
Found 21 abstracts in PubMed, retrieved 05.  
 

 What does Google say? 
 PDF only 
| .edu only 

---

**Interactive relationship** (e.g. stimulation, inhibition, etc)

**Stimulatory relationship**- The uncoupled proline substitutions in  **BtuB**  markedly affected the pattern of disulfide bonding to  **TonB** , both increasing the extent of cross linking and shifting the pairs of residues that can be joined.  Ref: 11029413 J Bacteriol, 2000
- Site directed disulfide bonding in intact cells has been used to show that the Ton box of  **BtuB**  and residues around position 160 of  **TonB**  approach each other in a highly oriented and specific manner to form  **BtuB**   **TonB**  heterodimers that are stimulated by the presence of transport substrate.  Ref: 11029413 J Bacteriol, 2000
**Neutral relationship**- Sequence changes in the ton box region of  **BtuB**  affect its transport activities and interaction with  **TonB**  protein.  Ref: 11029413 J Bacteriol, 2000
- This interaction with  **TonB**  occurs in part through an N terminal segment in the  **BtuB**  sequence called the Ton box.  Ref: 11705387 Biochemistry, 2001
- Uptake of cobalamins by the transporter protein  **BtuB**  in the outer membrane of Escherichia coli requires the proton motive force and the transperiplasmic protein  **TonB** .  Ref: 11029413 J Bacteriol, 2000
- The bacterial outer membrane transporter for vitamin B 12,  **BtuB** , derives its energy for transport by interacting with the trans periplasmic membrane protein  **TonB** .  Ref: 11705387 Biochemistry, 2001
- The Ton box sequence near the amino terminus of  **BtuB**  is conserved among all  **TonB**  dependent transporters and is the only known site of mutations that confer a transport defective phenotype which can be suppressed by certain substitutions at residue 160 in  **TonB** .  Ref: 11029413 J Bacteriol, 2000

**Non-interactive relationship** (e.g. studied together, co-existance, homology, etc.)

- Cross linking of  **BtuB**  and  **TonB**  in the presence of the  **BtuB**  V10P substitution became independent of the presence of substrate, indicating an additional distortion of the exposure of the Ton box in the periplasmic space.  Ref: 11029413 J Bacteriol, 2000
- The defect in cobalamin utilization resulting from the L8P substitution was suppressed by cysteine substitutions in adjacent residues in  **BtuB**  or in  **TonB** .  Ref: 11029413 J Bacteriol, 2000
- Transport of CN Cbl across the outer membrane and its accumulation in the periplasm is mediated by the  **TonB**  dependent transporter  **BtuB** .  Ref: 11790740 J Bacteriol, 2002
- We consider a variety of different colicins, including A, B, D, E1, E3, Ia, M and N, that penetrate through the porins OmpF, FepA,  **BtuB** , Cir and FhuA, to subsequently interact with a few targets in the periplasm, including TolA, TolB, TolC and  **TonB** .  Ref: 12423783 Biochimie< MedlineTA>Biochimie, 2002
